# Supplementary material for: Molecular characterization of antimicrobial resistance related genes in E. coli, Salmonella and Klebsiella isolates from broilers in the West Region of Cameroon
Source: PLoS One. 2023 Jan 11;18(1):e0280150. doi: 10.1371/journal.pone.0280150 (PMC9833522; doi:10.1371/journal.pone.0280150)
Supplement: S1 Appendix — (PDF) [file pone.0280150.s001.pdf]

S1 Appendix: Research Authorisation

REPUBLIQUE DU CAMEROUN
Paix-Travail-Patrie

REGION DE L'OUEST

LEGATION REGIONAL DE L'ELEVAGE, DES PECHES ET DES INDUSTRIES ANIMALES

SERVICE REGIONAL DES AFFAIRES GENERALES

BP 168 TEL : 233 44 40 46/233 44 14 92
FAX : 233 44 40 46

REPUBLIQUE OF CAMEROUN
Peace-Work-Fatherland

WEST REGION

REGIONAL DELEGATION OF LIVESTOCK, FISHERIES AND ANIMAL INDUSTRIES

REGIONAL SERVICE OF GENERAL AFFAIRS

N°...62.../18/L/DREPIA-O/SRAG

Bafoussam le, 04 JUIN 2018

Le Délégué Régional de l'Elevage, des Pêches et des Industries Animales de l'Ouest
Aux
Délégués Départementaux de l'Elevage, des Pêches et des Industries Animales de la Mifi, Koung-khi, Hauts-Plateaux, Nde, Menoua, Haut-Nkam, Bamboutos et Noun

Objet : Recherche pour la préparation d'une Thèse de Doctorat en Biochimie

Mesdames et Messieurs les Délégués départementaux,

Faisant suite à la demande de mise en stage 41/S /18/UdS/FS/DBC, pour le compte de Monsieur **LEINYUY JUDE FONBAH, Mle N°CM04-07SCI0836**, étudiant en Doctorat I de Biochimie à l'université de Dschang, introduite par le Chef de département par Intérim, pour effectuer des recherches portant sur le thème « **Identification phénotypique et biochimique et la détermination des mécanismes moléculaires de résistance aux antibiotiques des entérobactéries chez les poulets de chairs des fermes avicoles dans la Région de l'Ouest du Cameroun**» dans les Départements de la Mifi, Koung-khi, Hauts-Plateaux, Nde, Menoua ,Haut-Nkam, Bamboutos et Noun pour une période d'un an allant de Juin 2018 jusqu'en Juin 2019.

J'ai l'honneur de vous demander d'accueillir l'intéressé et de l'accompagner dans son domaine de recherche en vue de l'obtention de son doctorat en Biochimie.

Je vous saurai gré de l'accueil que vous réserverez à cet étudiant.

AMPLIATIONS :

- *INTERESSE*
- *CHRONO/ARCHIVES*

LE DELEGUE REGIONAL,

Dr Idrissou Baptist
Vétérinaire Hors -Echelle

This translates as:

REPUBLIC OF CAMEROON
Peace – Work – Fatherland
WEST REGION
REGIONAL DELEGATION OF LIVESTOCK, FISHERIES AND ANIMAL INDUSTRIES
REGIONAL SERVICE OF GENERAL AFFAIRS
P.O. Box 168: Tel: 233 44 40 46/233 44 14 92
Fax: 233 44 40 46
Number: 68/18/L/DREPIA-O/SRAG

Bafoussam, the 4th June 2018

The Regional Delegate for Livestock, Fisheries and Animal Industries for the West

To

Divisional Delegates for Livestock, Fisheries and Animal Industries for Mifi, Koung-khi, Hauts-Plateaux, Ndé, Menoua, Haut-Nkam, Bamboutos and Noun.

Subject: Research in preparation of a PhD thesis in Biochemistry

Dear Divisional Delegates,

Following the research request number 41/S/18/UDs/FS/DBC, on behalf of Mr. **LEINYUY JUDE FONBAH, Registration number CM04-07-SCI0836**, Doctorate Level I student in Department of Biochemistry at the University of Dschang, introduced by the Acting Head of Department, to carry out research on the theme **"phenotypic and biochemical identification, and the determination of molecular mechanisms of resistance to antibiotics in *Enterobacteriaceae* from broilers from poultry farms in the West Region of Cameroon"** in the Mifi, Koung-khi, Hauts-Plateaux, Ndé, Menoua, Haut-Nkam, Bamboutos and Noun Divisions for a period of one year from June 2018 to June 2019,

It is with honour that I ask you to welcome the concerned and to support him in his field of research with a view to obtaining his Doctorate Degree in Biochemistry.

I would appreciate your welcome to this student.

Signed: The Regional Delegate

Copied:

- ➢ **The Concerned**
- ➢ **Chrono/archives**

Informed Consent Statement: Consent by poultry owners to sample broilers was verbal after presentation of researcher credentials, research authorization and explanation of the work and the sampling procedure.
