# Supplementary material for: Molecular characterization of antimicrobial resistance related genes in E. coli, Salmonella and Klebsiella isolates from broilers in the West Region of Cameroon
Source: PLoS One. 2023 Jan 11;18(1):e0280150. doi: 10.1371/journal.pone.0280150 (PMC9833522; doi:10.1371/journal.pone.0280150)
Supplement: Attachment — Submitted filename: review report.docx [file pone.0280150.s002.docx]

This study highlights the importance of molecular methods in studying the dissemination of a particular resistance determinant in both resistant and susceptible isolates. I have only minor comments

Major comments:

1. In the Financial disclosure section, the authors mentioned that “The funders had no role in study design, data collection and analysis, decision to publish, or preparation of the manuscript”. Please provide the details related to the fund as per the author’s guideline.
2. In ethics statement section, the authors need to mention the approval number, type of consent –oral or written, etc.
3. In abstract, the methods part is missing. The main methods should be mentioned in the abstract section.

Minor comments:

Please correct the grammatical and typing errors in the text such as

1. In introduction section, last paragraph:

“We focused on 3 genera o……………………….. which known for the extensive colonisation of their guts by a myriad of Enterobacteriaceae”

Please rearrange the sentence

1. Please italicize the genus and species of bacteria throughout the text. For eg, in methods section, E.coli should be italicized.
