# Supplementary material for: Molecular characterization of antimicrobial resistance related genes in E. coli, Salmonella and Klebsiella isolates from broilers in the West Region of Cameroon
Source: PLoS One. 2023 Jan 11;18(1):e0280150. doi: 10.1371/journal.pone.0280150 (PMC9833522; doi:10.1371/journal.pone.0280150)
Supplement: Attachment — Submitted filename: Response to Reviewers.docx [file pone.0280150.s003.docx]

**Reviewer comments**

This study highlights the importance of molecular methods in studying the dissemination of a particular resistance determinant in both resistant and susceptible isolates. I have only minor comments

**Major comments:**

1. In the Financial disclosure section, the authors mentioned that “The funders had no role in study design, data collection and analysis, decision to publish, or preparation of the manuscript”. Please provide the details related to the fund as per the author’s guideline.

*The authors declared at submission while requesting for country-based waiver of publication fees, that the research was not externally funded. Resources for the research were provided by the authors. So we attached the letter from the Head of Department to that effect.*

*The financial disclosure statement selected might have been a submission error which has now been corrected in the resubmission.*

2. In ethics statement section, the authors need to mention the approval number, type of consent –oral or written, etc.

*In Cameroon, the only well-established institutional review board deals with ethical clearance of research on human subjects (the National Ethical Committee for Research involving Human Subjects – CNERSH/SP – Yaoundé, Cameroon). However, at the level of the universities, research projects dealing with animals are reviewed by experts and the protocol is submitted for administrative authorisation from the Ministry of Livestock, Fisheries and Animal Husbandry with a recommendation from the Head of Department concerned.*

*In addition, Law number 2021/014 of 09/07/2021 regulating access and use of genetic material and its derivatives in the Cameroonian territory in its article 4 is not binding to biological resources whose usage is not aimed at exploiting the genetic resources, as the case with our research (*[*https://www.prc.cm/en/news/the-acts/laws/5293*](https://www.prc.cm/en/news/the-acts/laws/5293)*).*

*A statement on the consent from farmers to sample broilers at farms has been included in the method section lines 101 to 104.*

3. In abstract, the methods part is missing. The main methods should be mentioned in the abstract section.

*The methods part in the Abstract has been updated to be explicit from lines 19 to 25.*

**Minor comments:**

Please correct the grammatical and typing errors in the text such as

1. In introduction section, last paragraph:

“We focused on 3 genera o……………………….. which known for the extensive colonisation of their guts by a myriad of Enterobacteriaceae”

*The sentence has been rearranged to bring out the intended meaning, see lines 71 to 74.*

2. Please italicize the genus and species of bacteria throughout the text. For eg, in methods section, E.coli should be italicized.

*The manuscript has been thoroughly reviewed and scientific names italicised.*

***Additional remarks***

1. Please ensure that your manuscript meets PLOS ONE's style requirements, including those for file naming.

*The manuscript has been thoroughly updated to meet journal’s specifications*

2. Please include your full ethics statement in the ‘Methods’ section of your manuscript file. In your statement, please include the full name of the IRB or ethics committee who approved or waived your study, as well as whether or not you obtained informed written or verbal consent. If consent was waived for your study, please include this information in your statement as well.

*We refer the Reviewer to previous comments in response to this concern (number 2 above)*

3. We note you have included a table to which you do not refer in the text of your manuscript. Please ensure that you refer to Table 1,2,5,6,7,9,10 & 11 in your text; if accepted, production will need this reference to link the reader to the Table

*We have made sure In-text table citations have been included as recommended*

4. We suggest you thoroughly copyedit your manuscript for language usage, spelling, and grammar. If you do not know anyone who can help you do this, you may wish to consider employing a professional scientific editing service.

*The manuscript has been thoroughly proofread and language usage, spelling, and grammar errors corrected*

5. Please review your reference list to ensure that it is complete and correct.

*The references have been re-checked to ensure that they match the journal’s specifications*

**Editor recommendation for title change**

Title change suggested may be "Molecular characterization of AMR related genes in *E. coli*, *Klebsiella* and *Salmonella* isolates from Cameroon”

*The authors feel that changing the title may in part mask the main aim of the research which not only pointed to the problem of resistance but raises the hypothesis of a mutated (resistance) gene (which does not necessarily translate to being resistant as detected in in-vitro AST) to contribute to the fate of the isolate (penetrance) and hence the ability to silently circulate in the study area.*
