# Supplementary material for: Molecular characterization of antimicrobial resistance related genes in E. coli, Salmonella and Klebsiella isolates from broilers in the West Region of Cameroon
Source: PLoS One. 2023 Jan 11;18(1):e0280150. doi: 10.1371/journal.pone.0280150 (PMC9833522; doi:10.1371/journal.pone.0280150)
Supplement: Attachment — Submitted filename: Response to Reviewers.docx [file pone.0280150.s004.docx]

**Response to Editor Comments**

Abstract section, methodology, poorly written, recheck spelling, simplify language, cut short abstract section!

*The methodology in the abstract has now been rewritten clearly, however much could not be done to contract the section without leaving out essential information. Lines 21 – 27.*

In result section, categorize, clearly mention number of susceptible isolates by disc diffusion method and minimum inhibitory concentration of each antibiotic used during susceptibility determination!

*The result section has been clearly categorised and the presentation of the tables simplified. In addition, The MIC of the antibiotics used can be found in line 151, Table 1.*

Mention name of *Salmonella* serovars, was all Typhi?

*The Salmonella serovars used have been specified. Line 112.*

Plasmid encoded AMR genes in *Salmonella*, *E. coli*, and *Klebsiella* are not characterized with proper protocol!

*Authors noted some commissions in the protocol and thank the reviewer for bringing this to our attention. We have now corrected this, mentioning clearly the necessary conditions, quantities and concentrations required for independent reproducibility of the resistance genes characterisation. Lines 161 – 171.*

Revise result presentation!

*The result section has been thoroughly revised.*

Conclusion: poorly written provide result based conclusion

*The conclusions have been reworded to reflect the primary findings of this work. This can be found in Lines 341 – 348.*

Change title as suggested previously by editor, as editor is not convinced with conclusion of penetrance! remove the section.

*The title has been modified to “****Molecular characterization of antimicrobial resistance related genes in E. coli, Salmonella and Klebsiella isolates from the West Region of Cameroon”*** *as suggested by the Editor.*

*Authors used the notion of penetrance here to made adequacy between genotype and phenotype. Though the expression of antibiotic resistance can vary in intensity depending on prevailing conditions, we used the notion of penetrance by extrapolation based the interpretations of disc diffusion AST only to give an idea of the proportion of isolates considered susceptible that will carry resistance genes.*
