# Supplementary material for: Molecular characterization of antimicrobial resistance related genes in E. coli, Salmonella and Klebsiella isolates from broilers in the West Region of Cameroon
Source: PLoS One. 2023 Jan 11;18(1):e0280150. doi: 10.1371/journal.pone.0280150 (PMC9833522; doi:10.1371/journal.pone.0280150)
Supplement: Attachment — Submitted filename: Response to Reviewers.docx [file pone.0280150.s005.docx]

**Response to reviewers**

**Journal Requirements**

- If you would like to make changes to your financial disclosure, please include your updated statement in your cover letter. Guidelines for resubmitting your figure files are available below the reviewer comments at the end of this letter.

*The financial disclosure provided remains unchanged.*

- If applicable, we recommend that you deposit your laboratory protocols in protocols.io to enhance the reproducibility of your results. Protocols.io assigns your protocol its own identifier (DOI) so that it can be cited independently in the future.

*No special protocol was developed by the authors in this manuscript.*

- Please review your reference list to ensure that it is complete and correct. If you have cited papers that have been retracted, please include the rationale for doing so in the manuscript text, or remove these references and replace them with relevant current references.

*The reference list has been thoroughly reviewed and none of the articles, web pages, and videos cited has been retracted. No modification has been made on the references.*

- While revising your submission, please upload your figure files to the Preflight Analysis and Conversion Engine (PACE) digital diagnostic tool, https://pacev2.apexcovantage.com/. PACE helps ensure that figures meet PLOS requirements.

*The manuscript does not contain images.*

**Additional Editor Comments**

- In the abstract section word treat be replaced by word threat!

*We thank the Editor for pointing out this spelling error which escaped out notice despite several revisions. Line 14. To fish out such errors, the manuscript has been submitted to a spelling and grammar check web site (*[*https://quillbot.com*](https://quillbot.com)*) and necessary corrections have been made.*

- Salmonella serovars names either S.Tphi or S.ParatyphiA or S.Paratyphi B or S.Paratyphi C and their sucesptibility to tested antibiotics be presented separately not as Salmonella spp.

*We have revised the research data to comply with the revision requested. Lines 113, 119, 181, and 196.*
